# Securin Is Not Required for Chromosomal Stability in Human Cells

**DOI:** 10.1371/journal.pbio.0030416

**Published:** 2005-11-29

**Authors:** Katrin Pfleghaar, Simone Heubes, Jürgen Cox, Olaf Stemmann, Michael R Speicher

**Affiliations:** **1**Institute of Human Genetics, Technical University Munich, Munich, Germany,; **2**Institute of Human Genetics, GSF National Research Center for Environment and Health, Neuherberg, Germany,; **3**Department of Molecular Cell Biology, Max Planck Institute of Biochemistry, Martinsried, Germany,; **4**Genedata GmbH, Martinsried, Germany; Stowers Institute for Medical ResearchUnited States of America

## Abstract

Abnormalities of chromosome number are frequently observed in cancers. The mechanisms regulating chromosome segregation in human cells are therefore of great interest. Recently it has been reported that human cells without an *hSecurin* gene lose chromosomes at a high frequency. Here we show that, after *hSecurin* knockout through homologous recombination, chromosome losses are only a short, transient effect. After a few passages *hSecurin^−/−^* cells became chromosomally stable and executed mitoses normally. This was unexpected, as the securin loss resulted in a persisting reduction of the sister-separating protease separase and inefficient cleavage of the cohesin subunit Scc1. Our data demonstrate that securin is dispensable for chromosomal stability in human cells. We propose that human cells possess efficient mechanisms to compensate for the loss of genes involved in chromosome segregation.

## Abstract 2

Speicher et al. show that previously reported chromosome instability and loss due to Securin gene knockout is a transient effect; human cells may have compensatory mechanisms to overcome Securin gene loss.

## Introduction

A number of factors are involved in ensuring that in dividing cells chromosomes are copied exactly once and then distributed correctly to daughter cells. Chromosome cohesion is established during chromosome replication in S-phase and is mediated by the multisubunit cohesin complex, which forms a giant ring structure possibly encircling sister chromatids [1]. Sister chromatid separation in anaphase depends on the removal of cohesin complexes from chromosomes [2]. In vertebrates removal of cohesin from chromosomes occurs in at least two steps. The “prophase pathway” removes the bulk of cohesin from chromosome arms during prophase and prometaphase [3,4]. By metaphase only minor amounts of cohesin remain on chromosomes, preferentially at centromeres [4]. Centromere-specific factors, such as shugoshin, protect the cohesion between sister centromeres from the prophase pathway [5,6]. At the metaphase-to-anaphase transition, residual cohesion is dissolved by the large cysteine endopeptidase separase, which cleaves the so-called kleisin subunit of cohesin (Scc1/Rad21 in mitosis; Rec8 in meiosis). This cleavage allows sister chromatids to move apart [7,8] and is, in fact, essential for anaphase to occur [9].

For most of the cell cycle, separase activity is inhibited by binding of an inhibitory chaperone called securin [10–12] or by phosphorylation-dependent complex formation with Cdk1 [13,14]. Separase is eventually activated by proteolysis of securin or the cyclin B subunit of Cdk1, which in both cases is mediated by a ubiquitin protein ligase named anaphase promoting complex or cyclosome (APC/C) and its cofactor Cdc20 [15,16].

Thus, securin is a key substrate of the APC/C^CDC20^ pathway. Though conserved in function, securins from different phyla are highly divergent in sequence [17]. Earlier studies had already implicated securin in functional mechanisms related to cell-cycle control and tumorigenesis [18,19]. To further address securin's function, both copies of the gene encoding *hSecurin* were inactivated via homologous recombination in the karyotypically stable human colorectal cancer cell line HCT116 [20]. The results indicated that *hSecurin* is indeed needed for chromosomal stability in human cells, as *hSecurin*-deficient cells exhibited high rates of chromosome missegregation, similar to those observed in many cancers. Furthermore, the data suggested that *hSecurin,* through its chaperone activity, plays a crucial role for the proper function of separase, especially for separase-dependent cleavage of the cohesin subunit Scc1 [20]. (Our group contributed to that paper some of the cytogenetic data using fixed cell suspensions provided by C. Lengauer's laboratory.)

However, the important role of *hSecurin* elucidated in the Jallepalli et al. study [20] contrasts with the results of another investigation, which found mice lacking securin to be viable and apparently normal [21]. Furthermore, only 20% of mouse cells without securin exhibit gains or losses of chromosomes [22].

To resolve this discrepancy, we conducted further studies with the *hSecurin^−/−^* cell line. Here, we show that the initial missegregation phenotype was superseded by a regaining of chromosomal stability in only a few passages. The karyotype of chromosomally stable *hSecurin^−/−^* cells was indistinguishable from that of the parent cell line with intact securin. Surprisingly, the initially described biochemical defects caused by the lack of securin, i.e., significantly reduced levels of separase and inefficient cleavage of the cohesin subunit Scc1 [20], were still present.

This indicates that securin is not required for faithful chromosome segregation and that alternative mechanisms may compensate for the absence of securin and/or reduced separase levels.

## Results

### Analysis of Chromosomal Instability in the *hSecurin^−/−^* Cell Line at Different Passages

In an initial step, metaphase spreads of the *hSecurin^−/−^* cell line were karyotyped by multiplex fluorescence in situ hybridization (M-FISH) at different passages (Figure 1). For passages 2 and 3, we confirmed the loss of numerous chromosomes in the majority of analyzed cells (Figure 1A and 1B). About 60% (12/20) of metaphase spreads showed losses of at least one chromosome. Surprisingly however, the high rate of chromosome losses in the *hSecurin^−/−^* cell line had almost vanished by passage 8 (Figure 1C), when chromosome losses were noted in only 10% (2/20) of cells. By passage 12, we observed no chromosome losses (Figure 1D). In the latter two analyses, merely one metaphase spread each had a gain of a single chromosome (Figure 1C and 1D).

**Figure 1:**
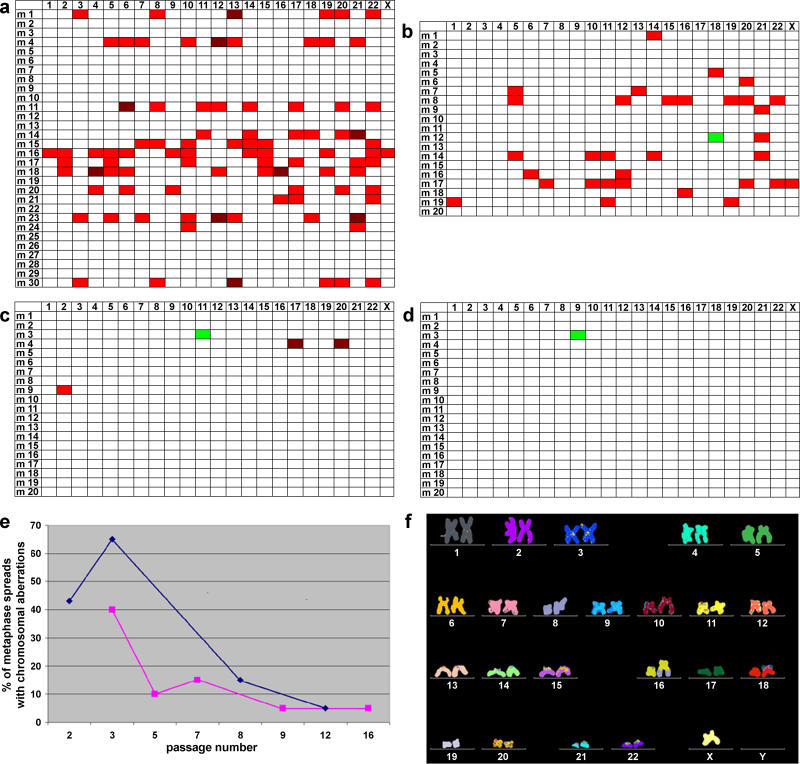
hSecurin^−/−^ Cells Regain Chromosomal Stability Quickly after *hSecurin* Knockout by Homologous Recombination: Summary of M-FISH Analysis of *hSecurin^−/−^* Cells at Different Passages (A–D) Graphic summary of M-FISH data from *hSecurin^−/−^* cells at passages 2 (A), 3 (B), 8 (C), and 12 (D). At each passage point 20 or 30 metaphase spreads were painted by M-FISH and analyzed for alterations of chromosome structure and number. Loss of a single copy of a given chromosome is marked in red, loss of both copies is marked in crimson, and gain of a single chromosome is marked in green. Rows indicate the analyzed metaphase spreads (m1 to m30 or m20); columns indicate the chromosome number (1–22 and X). (E) Graphic representation of the percentages of metaphase spreads with chromosomal copy number aberrations at different passages for the series of experiments shown in (A–D) (blue line) and for a repeat experiment (purple line). (F) M-FISH karyotype of a passage 12 *hSecurin^−/−^* cell, showing that the karyotype is identical to that of the parent cell line HCT116 (for details, see text).

The entire experiment was repeated, and it again showed the same phenomenon, i.e., decreasing chromosome losses in the *hSecurin^−/−^* cell line with increasing passage numbers (Figure 1E).

As expected, the parent cell line HCT116 was chromosomally stable and remained stable throughout all analyses (unpublished data).

When we karyotyped the *hSecurin^−/−^* cells at passage 12, we found that the karyotype of the cells was identical to the karyotype of the parent cell line HCT116 (Figure 1F; a detailed karyotype description is given in Materials and Methods). Thus, the *hSecurin^−/−^* cells showed all known structural rearrangements from the parental cell line HCT116, which we had described before [20,23]. There were no additional, new changes, and none of the known aberrations was lost.

Interphase FISH confirmed that *hSecurin^−/−^* cells at passage 12 were indeed chromosomally stable, similarly to the parental cell line HCT116 (Figure 2).

**Figure 2:**
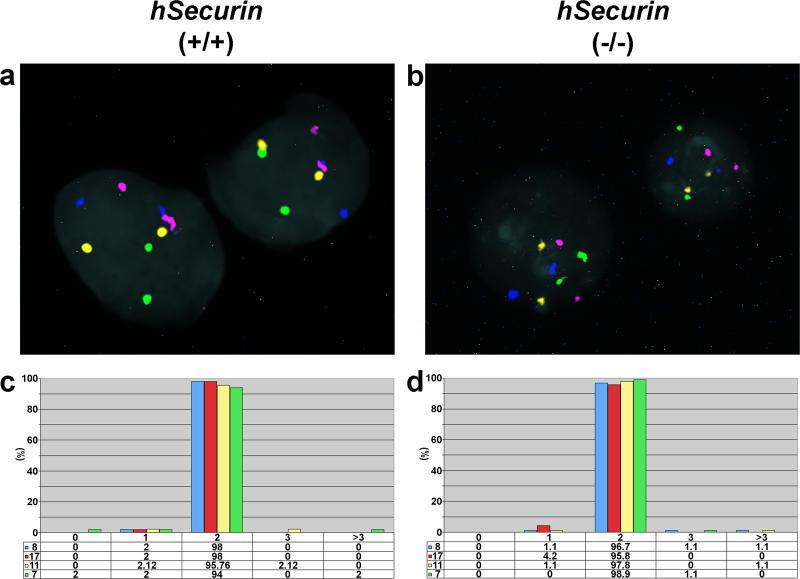
Assessment of Chromosomal Stability in Interphase Nuclei of Parental HCT116 Cells and Chromosomally Stable *hSecurin^−/−^* Cells Using Centromere-Specific Probes for Chromosomes 7, 8, 11, and 17 (A and B) Representative interphase FISH images of parental HCT116 (*hSecurin^+/+^*) (A) and *hSecurin^−/−^* (B) cell nuclei after hybridization of a four-color probe set consisting of centromere probes for chromosomes 7 (Cy5.5; purple), 8 (FITC; green), 11 (Cy5; blue), and 17 (Cy3; yellow). In each nucleus, two signals are visible for each probe. (C and D) Graphic summary of chromosome gains and losses in parental HCT116 (C) and *hSecurin^−/−^* (D) cells. The percentage of signals per nucleus for chromosomes 7, 8, 11, and 17 was determined from 300 cells of each genotype (100 cells each in three separate experiments).

### Confirmation that Chromosomally Stable Cell Lines Are Indeed *hSecurin^−/−^* Cells

In the next step we confirmed that chromosomally stable *hSecurin* ^−/−^ cells were indeed *hSecurin* deficient. We used PCR primer pairs spanning the second and third exons of the securin gene, as described previously [20] (Figure 3A and 3B). In addition, we designed a primer set flanking exon 3 to specifically demonstrate that the *hSecurin* ^−/−^ cells lack exon 3 of the securin gene (Figure 3A and 3C). Genomic PCR analyses with these primer sets using DNA extracted from chromosomally stable *hSecurin* ^−/−^ cells demonstrated that the cells had indeed a homozygous deletion of the exon 3 region of the *hSecurin* gene. Primers for exons 8 and 9 of p53 were used as an additional control (Figure 3B).

**Figure 3:**
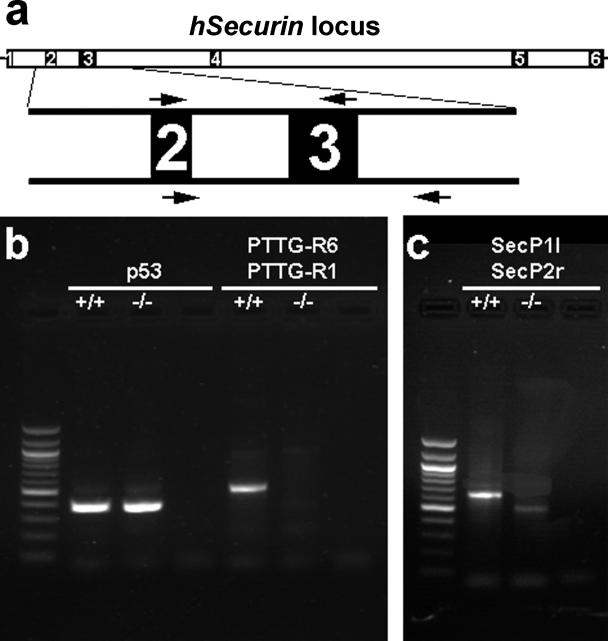
Verification that the Chromosomally Stable Cells Indeed Lack Part of *hSecurin* by Analyses of Genomic DNA from Parental HCT116 Cells (+/+) and Chromosomally Stable *hSecurin^−/−^* Cells (−/−) (A) Transcript structure of the *hSecurin* gene with its six exons. The lengths of introns and exons are drawn to scale based on the NCBI 35 assembly of the human genome (http://www.ensembl.org). Exons 2 and 3, with the locations of the respective primer pairs, are depicted enlarged. (B) As a control, PCR analysis was done with primers located in exons 8 and 9 of the p53 gene and resulted in the expected amplification product for both cell lines (lanes 2 and 3). In contrast, PCR with primers PTTG-R6 and PTTG-R1, located in the second and third exon of the *hSecurin* gene (arrows above exons 2 and 3 in [A]), yielded an amplification product only for the parental HCT116 cells (+/+; lane 5) and not for the chromosomally stable *hSecurin^−/−^* cells (−/−; lane 6). Lane 1 shows the 100-bp ladder as a size marker, and lanes 4 and 7 are negative controls for the respective primer pairs. (C) PCR analyses with primers SecP1l, located in exon 2, and SecP2r, located in intron 3–4 (arrows below exons 2 and 3 in [A]), resulted in amplification products with different sizes (lanes 2 and 3), reflecting the deletion of exon 3. Lane 1 shows the 100-bp ladder; lane 4 is the negative control.

### Normal Execution of Anaphase in *hSecurin^−/−^* Cells

Previously, it was reported that cells lacking *hSecurin* grew somewhat more slowly than wild-type cells [20]. In contrast, the growth pattern of chromosomally stable *hSecurin^−/−^* cells was indistinguishable from that of the HCT116 parent cell line (unpublished data). Therefore, we performed immunofluorescence experiments to examine the distribution of centromeres during mitosis with cells from passages 12 or higher. *hSecurin* ^+/+^ cells and chromosomally stable *hSecurin* ^−/−^ cells in various stages of mitosis were stained with the CREST (calcinosis-Raynaud's phenomenon-esophageal dismobility-sclerodactyly-telangiectasia syndrome of scleroderma) antibody, which recognizes kinetochore proteins (Figure 4). In addition, we used cyclin B1 as a marker for mitotic stage. Anaphase cells were first identified by virtue of chromosome condensation and lack of cyclin B staining and then scored for unsegregated chromatids remaining at the metaphase plate. In previous experiments, about 30% of *hSecurin* ^−/−^ cells in anaphase still had paired sister chromatids left behind at the metaphase plate, when most of the other chromosomes had segregated to the poles [20]. When we analyzed a total of 75 cells in three separate experiments in each *hSecurin* ^+/+^ (Figure 4A–4G) and *hSecurin* ^−/−^ (Figure 4H–4N) cell line, we found no differences. In fact, the vast majority of analyzed anaphase cells in each cell line displayed a complete separation of sister chromatids and migration of centromeres to opposite poles (Figure 4O).

**Figure 4:**
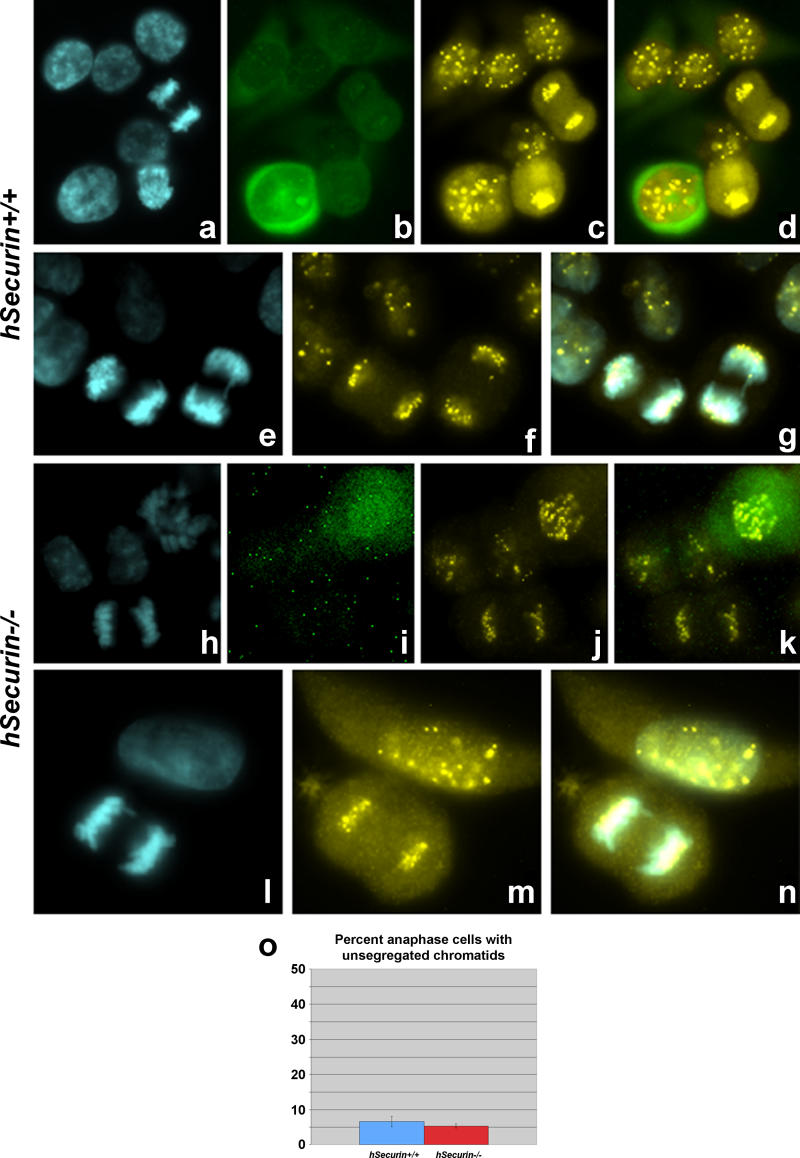
Analysis of Defective Sister Chromatid Separation in *hSecurin* ^−/−^ Cells *hSecurin* ^+/+^ cells and *hSecurin* ^−/−^ cells were stained with DAPI as a counterstain, a cyclin B1 antibody (green/FITC), which stains cells in the early mitosis but not in anaphase, and a CREST antibody (yellow/Cy3) to visualize kinetochores. In each image are telophase cells showing a complete separation of sister chromatids. (A–D) Analysis of several cells of the HCT116 parent cell line (*hSecurin* ^+/+^). The sequence of images illustrates the DAPI (A), FITC (B), and Cy3 (C) channels, while (D) shows the merged FITC and Cy3 images. One cell is stained with the cyclin B1 antibody; the majority of cells are in prophase (characteristic “double-dot” pattern of paired centromeres). There are two telophase cells. (E–G) Two telophase *hSecurin* ^+/+^ cells with complete separation of sister chromatids. (E) DAPI; (F) Cy3; (G) merged DAPI and Cy3 image. (H–K) Cells from the *hSecurin* ^−/−^ cell line. The cell in the upper-right corner is stained with the cyclin B1 antibody; at the bottom is a normal telophase cell with complete separation of sister chromatids. (H) DAPI; (I) FITC; (J) Cy3; (K) merged FITC and Cy3 image. (L–N) Prophase and telophase *hSecurin* ^−/−^ cells. The telophase cell demonstrates a complete separation of sister chromatids. (L) DAPI; (M) Cy3; (N) merged DAPI and Cy3 image. (O) Quantitation of the chromatid separation defect in *hSecurin* ^+/+^ and *hSecurin* ^−/−^ cells. The percentage of anaphase cells with unsegregated sister chromatids at the metaphase plate was determined from 75 cells of each genotype (25 cells each in three separate experiments; the error bars indicate standard deviation).

Furthermore, as in the first paper [20], we did not observe premature sister chromatid separation when cells were exposed to microtubule poisons such as colchecine (unpublished data).

These results indicate that chromosomally stable *hSecurin* ^−/−^ cells execute anaphase normally, with complete sister chromatid separation.

### Cleavage of Separase and Scc1 in *hSecurin^−/−^* Cells

During mitosis, separase undergoes proteolytic auto-cleavage resulting in carboxy (C)- and amino (N)-terminal fragments. Previously, it was reported that in *hSecurin* ^−/−^ cells separase levels and activity were both reduced [20].

We analyzed separase levels in chromosomally stable *hSecurin* ^−/−^ cells synchronized by nocodazole. Lysates from *hSecurin* ^+/+^ and *hSecurin* ^−/−^ cells were probed with antibodies to separase. For each cell line we detected both the full-length and the cleaved forms of separase (Figure 5A). However, both the full-length and the cleaved forms of separase were consistently 3- to 4-fold weaker in the chromosomally stable *hSecurin* ^−/−^ cells.

**Figure 5:**
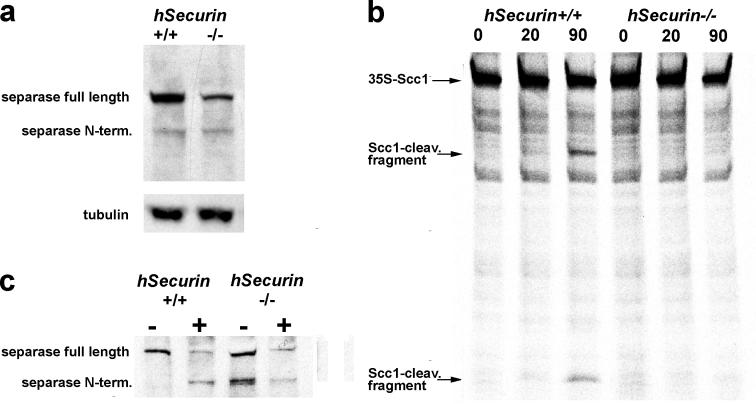
Chromosomally Stable *hSecurin* ^−/−^ Cells of Passage 12 and Higher Show Reduction in Both the Level and the Activity of Separase (A) Quantitation of full-length separase and the N-terminal cleavage product in both *hSecurin* ^+/+^ and *hSecurin* ^−/−^ cells. Lysates from nocodazole-arrested cells were analyzed by immunoblotting with an antibody against the N-terminus of separase. The chromosomally stable *hSecurin* ^−/−^ cells show reduced levels of both the full-length and the cleaved N-terminal form of separase. β-tubulin was used as a loading control. (B) Separase was immunoprecipitated from nocodazole-arrested *hSecurin* ^+/+^ and *hSecurin* ^−/−^ cells, activated by incubation in *Xenopus* anaphase extracts, and incubated with ^35^S-hScc1 for 0, 20, or 90 min before analysis by SDS-PAGE and autoradiography. For these experiments we used four times as many *hSecurin* ^−/−^ cells as *hSecurin* ^+/+^ cells. Note the absence of detectable Scc1 cleavage fragments in the *hSecurin* ^−/−^ samples. (C) Separase used for the activity assay in (B) was analyzed by Western blotting before (−) and after (+) exposure to *Xenopus* anaphase extracts. The *hSecurin* ^+/+^ cells clearly demonstrate an increase in self-cleavage of separase upon activation in the extract. Occurrence of auto-cleavage in *hSecurin* ^−/−^ cells even before incubation in *Xenopus* extract suggests deregulation of separase, at least under the given conditions of this in vitro experiment.

We reconstituted the cleavage reaction, which dissociates Scc1 from the centromeric regions, by using immunoprecipitated separase complexes that were first incubated with *Xenopus* egg extracts as a source of mitotic APC/C^CDC20^. In the case of HCT116 parent cells, incubation of activated separase with in vitro-translated ^35^S-hScc1 resulted in typical cleavage fragments that were readily detectable after a 20-min incubation (Figure 5B). Four times as much starting cell material was used to purify separase from *hSecurin* ^−/−^ cells as was used to purify the same amount of separase from wild-type cells (Figure 5C). However, the separase from *hSecurin* ^−/−^ cells did not display any cleavage activity towards Scc1, even after a 90-min incubation (Figure 5B).

Despite the absence of activity in vitro, separase auto-cleavage products (Figure 5A) demonstrate the presence of at least some separase activity in *hSecurin* ^−/−^ cells, which, apparently, is sufficient to execute anaphase normally (see above).

Interestingly, immunoprecipitated separase from nocodazole-arrested cells showed a higher degree of self-cleavage in *hSecurin* ^−/−^ cells compared to that in wild-type cells. This suggests that separase might be partly deregulated in the *hSecurin* ^−/−^ cells (Figure 5C).

## Discussion

We report here that *hSecurin* ^−/−^ cells are capable of compensating securin loss and rapidly regain chromosomal stability within a few passages. Our findings were unexpected, as chromosomally stable *hSecurin* ^−/−^ cells continue to have the initially described biochemical defect, i.e., reduction of both the amount and the activity of separase [20; this study].

Our data may explain why mice lacking securin are viable and normal [21]. However, mouse cells without securin have little change in the level of separase [22]. This is in contrast to our observations in human cells, where absence of securin resulted in the aforementioned significant reduction of both separase and its cleavage product. Furthermore, about 20% of mouse embryonic stem cells without securin were aneuploid [22], while the percentage of aneuploid human *hSecurin* ^−/−^ cells was reduced to background levels similar to those observed in the chromosomally stable parent cell line HCT116. These data indicate that significant differences in separase regulation between human and mouse cells must exist.

The reconstitution of chromosomal stability and complete separation of sister chromatids in *hSecurin* ^−/−^ cells suggest that significantly lower than normal amounts of separase are sufficient for normal execution of anaphase. This is in agreement with separase at wild-type levels being able to efficiently remove from chromosomes even vastly increased amounts of cohesin [7].

In contrast to budding yeast, human cells lacking *hSecurin* still manage to arrest sister chromatid separation in the presence of spindle poisons [20; unpublished data]. Therefore, additional mechanisms that regulate the removal of cohesin in human cells must exist. One additional, securin-independent mechanism of separase inhibition involves phosphorylation by Cdk1 and subsequent binding of the kinase [13,22]. As securin and Cdk1 bind separase in a mutually exclusive manner [14], Cdk1 may be capable of compensating for the loss of securin. Indeed, mouse embryonal stem cells lacking both forms of separase regulation suffer from precocious sister chromatid separation under mitotic checkpoint arrest [22]. Another level of control is probably exerted by shugoshin, which prevents removal of centromere-specific cohesin before the onset of anaphase [5].

Our findings demonstrate that deletion of *hSecurin* has little or no effect on long-term chromosome segregation fidelity in human cells. In fact, our results even raise the possibility that the chromosomal instability (CIN) phenotype observed in early passages of the *hSecurin* ^−/−^ cells might have been caused by insults during the deletion process rather than by loss of securin per se.

Alternatively, cells might upregulate other control pathways in response to loss of securin, thereby regaining chromosomal stability. Comparative gene expression profiling before and after loss of *hSecurin* might reveal compensatory changes in the expression of genes involved in anaphase regulation. We therefore compared the transcriptomes of the HCT116 parent and the *hSecurin* ^−/−^ cell line using the Affymetrix U133A chip. Indeed, significantly different expression levels were found for *PLK2* (Polo-like kinase 2), *RCC1* (regulator of chromosome condensation 1, also known as chromosome condensation 1 [*CHC1*]), and *SMC6L1* (SMC6 structural maintenance of chromosomes 6-like 1) (unpublished data). However, future experiments will be required to determine the physiological significance of these findings and whether the above proteins might play a currently unknown role in the other two known regulations of sister chromatid separation.

In summary, we have shown that securin is not required for chromosomal stability in human cells. Our results affect current mathematical models of colorectal cancer investigating the role of genetic instability in tumorigenesis [24]. The crucial effect attributed to CIN is acceleration of the mutation rate. However, our data indicated that a CIN-causing mutation may not reach fixation in a given cell compartment, which should therefore change existing assumptions on the evolution of CIN lesions and their growth rates.

Finally, implications of this study extend beyond mechanisms leading to chromosomal instability and affect possible strategies for cancer therapy. It has been suggested that, as stability pathways of tumor cells are defective, cancer cells may be more sensitive to stress-inducing agents and they should be especially susceptible to attack by instability-inducing drugs [25]. However, our results suggest that targeting a particular pathway may not destroy a cell but rather activate alternative pathways. A search and detailed characterization of these alternative pathways will provide further clues to the nature of CIN in human cancers.

## Materials and Methods

### Cell lines

The colorectal cell line HCT116 was used as parent cell line. This is a chromosomally stable cell line; the karyotype has been described before as 45,X,-Y,der(10)dup(10)(q24q26)t(10;16)(q26;q24), der(16)t(8;16)(q13;p13),der(18)t(17;18)(q21;p11.3) [20,23]. The *hSecurin^−/−^* cell line was generated by homologous recombination as described previously [20]. Early passage stocks of both cell lines were generously provided by C. Lengauer and B. Vogelstein (both Johns Hopkins Oncology Center, Baltimore, Maryland, United States).

### Culturing of parent and *hSecurin* knockout cells

HCT116 cells and HCT116 *hSecurin* ^−/−^ cells were cultured in McCoy's 5A medium (Gibco Invitrogen, Karlsruhe, Germany) supplemented with 10% fetal bovine serum (FBS) (Biochrom, Berlin, Germany), 100 units/ml penicillin, and 0.1 mg/ml streptomycin. Monolayer cultures were grown at 37 °C in a 5% CO_2_ atmosphere and were split 1:3 twice a week.

### M-FISH and interphase FISH

M-FISH [26] was done with 7-fluorochromes as described previously [27]. For interphase FISH we used centromere probes, which were generously provided by M. Rocchi (for detailed information, see http://www.biologia.uniba.it/rmc/index.html). We assembled a four-centromere probe set consisting of centromere-specific probes for chromosomes 7 (PZ7.6B; indirectly labeled with Cy5.5), 8 (PZ8.4; directly labeled with FITC), 11 (PRB11; directly labeled with Cy5), and 17 (PZ17–14; directly labeled with Cy3).

### PCR verification of *hSecurin* knockout

To verify that part of the *hSecurin* locus was deleted by homologous inactivation, we used the same four PCR primers as previously described [20]: two primers located in exon 2 (PTTG-R6 [AAAATGGAGAACCAGGCACC] and PTTG-gen01 [ACCCGTGTGGTTGCTAAGGA]) and two primers within exon 3 (PTTG-R1 [GGTCCCTTGGTCTTTACAGA] and PTTG-R4 [GTGGGCATCGAACGTTTTG]). These primers define two STS markers of the *hSecurin* locus that were homozygously deleted in *hSecurin^−/−^* cells [20]. In addition, we used the following set of primers to specifically demonstrate that *hSecurin^−/−^* cells lack exon 3 of the *hSecurin* gene: SecP1l (GATGGGCTGAAGCTGGG), which is located in exon 2, and SecP2r (TGCTTGCTAACCTCTATTTCCC), which is within intron 3–4.

As an additional control we used primers for exons 8 and 9 within *TP53*. The 3′ primer was CATGATTCAGAACCCTGGAG; the 5′ primer was AGGACCTGATTTCCTTACTGC.

### Analysis of sister chromatid separation

Cells were grown on coverslips for 24 h in McCoy's 5A medium (Gibco Invitrogen) plus 100 units/ml penicillin and 0.1 mg/ml streptomycin without FBS. Aphidicolin was added to a final concentration of 0.15 μg/ml in McCoy's 5A medium with 10% FBS. After 14 h, the medium was removed, and cells were washed four times with PBS and cultured for 8–10 h in McCoy's 5A medium plus 10% FBS to obtain cells in anaphase.

Cells were fixed in a 4% paraformaldehyde solution, washed three times with PBS/0.2% Tween, and permeabilized in 0.5% TritonX in PBS/Tween for 15 min.

After being blocked with 4% BSA in PBS/Tween, cells were incubated with CREST serum (Euroimmun Corp., Gross Groenau, Germany) in a 1:100 dilution.

After being washed as above, cells were incubated with rabbit anti-human antibody conjugated with Cy3 (Dianova GmbH, Hamburg, Germany) and anti-cyclin B1 monoclonal antibody (Santa Cruz Biotechnology, Santa Cruz, California, United States). After being washed as above, cells were counterstained with DAPI and mounted in antifade for fluorescence microscopy.

### Separase quantification by immunoblotting

HCT116 wild-type and *hSecurin^−/−^* cells were grown in McCoy's medium (10% FBS, 100 units/ml penicillin, 0.1 mg/ml streptomycin) and synchronized by addition of nocodazole (0.2 μg/ml final concentration) for 14 h. Cells from six 75-ml dishes (70% confluent) of each cell line were lysed in 1 ml of 20 mM Tris/HCl (pH 7.7), 100 mM NaCl, 10 mM NaF, 20 mM β-glycerophoshate, 5 mM MgCl_2_, 0.1% Triton X100, 5% glycerol, 1 μM microcystin-LR, and Complete protease inhibitors (Roche, Basel, Switzerland). After ultracentrifugation at 100,000 g, supernatants were analyzed by Western blotting using an antibody directed against the N-terminus of separase [13]. Signals were quantified by normalizing to β-tubulin (monoclonal antibody obtained from the Developmental Studies Hybridoma Bank, Iowa City, Iowa, United States).

### Separase activity assay

HCT116 wild-type (four 75-ml dishes) and *hSecurin^−/−^* cells (12 75-ml dishes) were lysed as above, and separase was immunoprecipitated with a rabbit polyclonal antibody raised against the sequence GSDGEDSASGGKTPA of human separase. For each immunoprecipitation, 8 μg of antibodies was prebound to 30 μl of Protein G Sepharose 4 Fast Flow (Amersham Biosciences, Little Chalfont, United Kingdom). Separase activation in *Xenopus* extract and Scc1 cleavage assays were done as described elsewhere [13], except that the Scc1 cleavage reaction was performed in the presence of 1.3 μg/μl antigenic peptide. Amounts and self-cleavage of separase were analyzed by immunoblotting aliquots before and after incubation in the extract.

## Abbreviations

M-FISH: multiplex fluorescence in situ hybridization
